# How to assess the impact of diagnosis‐to‐ablation time in patients with early‐onset atrial fibrillation

**DOI:** 10.1002/clc.24226

**Published:** 2024-02-02

**Authors:** Naoya Kataoka, Teruhiko Imamura

**Affiliations:** ^1^ Second Department of Internal Medicine University of Toyama Toyama Japan

To the Editor,

Zhou et al. have elucidated that a reduced diagnosis‐to‐ablation time correlates with diminished susceptibility to cardiovascular incidents and recurrent atrial fibrillation among patients exhibiting early‐onset atrial fibrillation.^1^ However, several pivotal concerns have been raised.

One significant query pertains to the rationale behind the authors' delineation of the threshold for early‐onset atrial fibrillation at 45 years,^1^ as opposed to the commonly established 60 years. Previous literature has indicated the conjunction of early‐onset atrial fibrillation with hypertension at the age of 40.^2^ This early‐onset manifestation of atrial fibrillation might potentially intertwine with distinctive genetic disorders.^3^ Notably, the recurrence rate of atrial fibrillation following catheter ablation stands at 40% over a span of 5 years,^1^ signifying a considerable rate warranting comprehensive interventions addressing associated comorbidities or underlying etiologies alongside atrial fibrillation ablation.

The protocol for managing recurrent atrial fibrillation remains nebulous. While the authors evaluated not only atrial fibrillation recurrence but also mortality,^1^ readmission due to heart failure, and incidences of bleeding, the strategy for addressing recurrent atrial fibrillation and its consequential impact on these outcomes necessitates elucidation.

Accurate diagnosis of concurrent heart failure in patients afflicted with atrial fibrillation poses a challenge due to their overlapping symptoms. Notably, 5% of subjects in the authors' study exhibited heart failure.^1^ Clarity regarding the authors’ methodology for diagnosing concurrent heart failure is imperative.

Furthermore, the authors' definition of cardiac rehospitalization lacks precision.^1^ It seemingly encompasses acute coronary syndrome, which may not inherently correlate with atrial fibrillation. A preferable approach might entail segregating heart failure rehospitalization to better discern the intricate association between atrial fibrillation and heart failure.

## CONFLICT OF INTEREST STATEMENT

The authors declare no conflicts of interest.
